# Electrochemical Activation of LiGaO_2_: Implications for Ga-Doped Garnet Solid Electrolytes in Li-Metal Batteries

**DOI:** 10.1021/acsami.4c03729

**Published:** 2024-07-16

**Authors:** Anna Windmüller, Kristian Schaps, Frederik Zantis, Anna Domgans, Bereket Woldegbreal Taklu, Tingting Yang, Chih-Long Tsai, Roland Schierholz, Shicheng Yu, Hans Kungl, Hermann Tempel, Rafal E. Dunin-Borkowski, Felix Hüning, Bing Joe Hwang, Rüdiger-A. Eichel

**Affiliations:** † Institute of Energy Technologies (IET-1: Fundamental Electrochemistry), Forschungszentrum Jülich, Jülich 52425, Germany; ‡ Department of Chemical Engineering, Nano-electrochemistry Laboratory, National Taiwan University of Science and Technology, Taipei City 106, Taiwan; § Sustainable Electrochemical Energy Development Center, National Taiwan University of Science and Technology, Taipei City 106, Taiwan; ∥ Ernst Ruska-Centre for Microscopy and Spectroscopy with Electrons (ER-C 1), Forschungszentrum Jülich GmbH, Jülich 52425, Germany; ⊥ Institute of Electrical Engineering and Information Technology, FH Aachen − University of Applied Sciences, Aachen 52066, Germany; # Institute of Physical Chemistry (IPC), RWTH Aachen University, Aachen 52066, Germany

**Keywords:** LiGaO_2_, garnet solid electrolyte, Ga-doping, Li_7_La_3_Zr_2_O_12_, solid-state battery

## Abstract

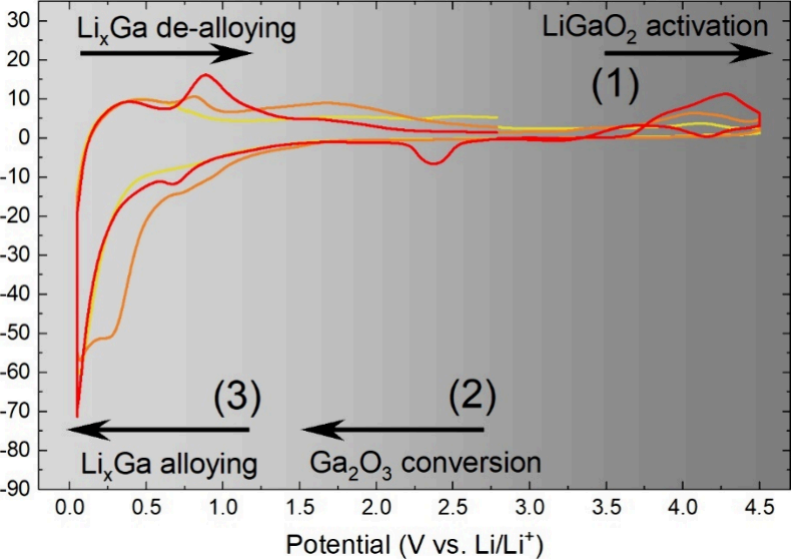

Ga-doped Li_7_La_3_Zr_2_O_12_ garnet solid electrolytes exhibit the highest Li-ion conductivities among the oxide-type garnet-structured solid electrolytes, but instabilities toward Li metal hamper their practical application. The instabilities have been assigned to direct chemical reactions between LiGaO_2_ coexisting phases and Li metal by several groups previously. Yet, the understanding of the role of LiGaO_2_ in the electrochemical cell and its electrochemical properties is still lacking. Here, we are investigating the electrochemical properties of LiGaO_2_ through electrochemical tests in galvanostatic cells versus Li metal and complementary *ex situ* studies via confocal Raman microscopy, quantitative phase analysis based on powder X-ray diffraction, energy-dispersive X-ray spectroscopy, X-ray photoelectron spectroscopy, and electron energy loss spectroscopy. The results demonstrate considerable and surprising electrochemical activity, with high reversibility. A three-stage reaction mechanism is derived, including reversible electrochemical reactions that lead to the formation of highly electronically conducting products. The results have considerable implications for the use of Ga-doped Li_7_La_3_Zr_2_O_12_ electrolytes in all-solid-state Li-metal battery applications and raise the need for advanced materials engineering to realize Ga-doped Li_7_La_3_Zr_2_O_12_for practical use.

## Introduction

Garnet-structured solid electrolytes enable all-solid-state Li-metal batteries (ASSLMBs) to have the potential to enhance the safety and energy density of ASSLMBs significantly in comparison to conventional Li-ion batteries (LIB).^1−3^ Among the family of garnet-structured solid electrolytes derived from Li_7_La_3_Zr_2_O_12_ (LLZO), Ga-doped LLZO (Ga:LLZO) shows the highest Li-ion conductivity at room temperature, exceeding 1 mS cm^–1^.^1,4,5^ Ga:LLZO thus receives a lot of interest and is intensely studied for its relation of crystal chemistries, structures and ionic conductivities,^6−11^ structural stabilization for optimizing its conductivities,^12,13^ sintering optimization for creating dense ceramics with high purity,^7,14−16^ thin film deposition for the application as thin film electrolyte,^17^ and application as a thick film for solid-state battery fabrications.^4,5,13,14^ In general, it has been found that optimized conductivities >1 mS cm^–1^ at room temperature can be achieved through the stabilization of the cubic garnet phase at low levels of Ga-doping, that is, 0.2 < *x* < 0.3 in Li_7–3*x*_Ga_*x*_La_3_Zr_2_O_12_, while 6.4 < Li < 6.6.^6,11,15,17^

For higher Ga substitutions (*x* > 0.5 mol per formula unit of LLZO), β-LiGaO_2_ emerges as an obvious secondary phase in Ga:LLZO that can be detected by X-ray diffraction^7,9,10^ and at the grain boundaries of the garnet phase by electron microscopy.^6^ However, studies employing transmission and electron microscopy have recently demonstrated that even at considerably low Ga-doping levels (<0.1 mol per formula unit of LLZO), LiGaO_2_ can be found at grain boundaries and in triple junctions in the densified Ga:LLZO ceramic pellets.^4,5,13^ At the same time, it has been reported that LiGaO_2_ accumulation on the grain boundary can be correlated to the instability of Ga:LLZO toward Li metal.^4,5,13^ For example, Li et al. reported severe cracking once Ga:LLZO pellet is in contact with molten Li.^13^ In an attempt to study the reaction mechanism, LiGaO_2_ was reacted with liquid Li metal, where Li_2_Ga and Li_2_O formation was observed. The huge volume expansion from these educts to products was thus identified as the cause of the microstructural cracking to collapse LiGaO_2_ containing Ga:LLZO pellets when in contact with molten Li metal.^13^

Various efforts were undertaken to control and eliminate the presence of LiGaO_2_ phases in Ga:LLZO to improve the stability of Ga:LLZO against Li metal effectively. Su et al. found that the presence of LiGaO_2_ is related to abnormal grain growth of LLZO particles during sintering.^16^ They developed a processing strategy via a two-step sintering leading to fine grains of Ga:LLZO after the sintering process and thus avoiding LiGaO_2_ secondary phase formation to improve the stability against Li metal.^16^ Li et al. reported to control and suppress the secondary LiGaO_2_ phase formation by adding 2 wt % SiO_2_ into Ga:LLZO powder during the sintering process.^13^ This resulted in improved thermomechanical and electrochemical stability when in contact with molten Li. It is hypothesized that SiO_2_ extracts Li from LiGaO_2_ to form Li_2_SiO_3_ while the remaining Ga incorporates into the LLZO bulk.^13^

In one of our previous studies, we compared and analyzed the reactions between Li metal and sintered Li_6.4_Ga_0.2_La_3_Zr_2_O_12_ and Li_6.45_Ga_0.05_La_3_Zr_1.6_Ta_0.4_O_12_, in comparison to Ga-free Li_6.45_Al_0.05_La_3_Zr_1.6_Ta_0.4_O_12_ (Ta:LLZO). We demonstrated that Ga:LLZO undergoes chemical reactions with Li metal, while Ta:LLZO remains stable. In this study, we could also demonstrate that Ga from the Ga:LLZO bulk forms Li–Ga alloys at the grain boundaries of the Ga:LLZO ceramic after reaction with molten Li. By NMR measurements, it was proven that Ga-ions can leach out from the bulk into the grain boundary after contacting Li metal.^4,5^ The primary observation of Ga leaching into the grain boundaries of the Ga:LLZO ceramic questions the actual role of LiGaO_2_ in the failure mechanism of the Ga:LLZO solid electrolyte.

In the literature, β-LiGaO_2_ is well-known in the area of semiconductor research and semiconductor industry, where it serves as a substrate for GaN growth.^18^ β-LiGaO_2_ itself can be grown into rather large single crystals,^18,19^ and its crystal structure was determined by Marezio in 1964 to be identical to that of orthorhombic β-NaFeO_2_.^20^ Therefore, its structural, optical, and electrical properties are well understood,^21−24^ but it is hard to extrapolate, which role LiGaO_2_ may play in the instability of Ga:LLZO toward Li, even in an isolated environment without physical contact to Li. Furthermore, its stability and reactions under a larger electrochemical voltage, such as in a full battery cell, are not yet understood. Here, we investigate LiGaO_2_ from an electrochemical point of view to understand the possible impact of β-LiGaO_2_ in Ga:LLZO ceramics under dynamic conditions, i.e., under an applied electrical field and electrochemical cycling conditions, as in a battery. To accomplish this, we synthesize β-LiGaO_2_ and examine it as the positive electrode in a galvanic cell for understanding its electrochemical properties. The results contribute to a better understanding of the Ga:LLZO failure mechanism when in contact with Li metal, which may offer a better strategy for fabricating and processing the Ga:LLZO solid electrolyte for ASSLMB application.

## Experimental Section

### LiGaO_2_ Synthesis and Electrode Preparation

An adapted version of the Ga:LLZO synthesis procedure was used to produce LiGaO_2_ powder.^4^ The synthesis was performed by mixing Li_2_CO_3_ and Ga_2_O_3_ powders in an autogrinder (Retsch) in a 1:1 ratio, adding a slight excess of Li_2_CO_3_(10 wt %) to compensate for Li-evaporation losses. The powder mixture was heated to a temperature of 1200 °C with a heating ramp of 5 K min^–1^ in a muffle furnace in air in a closed corundum crucible. The reaction product was cooled freely after 8 h and reground in the autogrinder to produce a fine powdered sample.

To test the powder for its electrochemical activity, it was processed by a typical laboratory-scale procedure into an electrode tape with a 10:10:80 ratio of polyvinylidene difluoride (PvDF, Alfa-Aesar): carbon black: LiGaO_2_.^13^ Prior to the slurry preparation, carbon black powder (Super P, Alfa-Aesar) and LiGaO_2_ powder were dried in a vacuum furnace at 80 °C for 16 h. The powder was mixed and ground in a mortar for more than 20 min. The mixture was added to polyvinylidene difluoride (PvDF), dissolved in *N*-methylpyrrolidone (NMP, Alfa-Aesar), and stirred vigorously for 5 h. The slurry was drop-coated on a ⌀11 mm Ni current collector and dried for at least 24 h in a hood, following another 24 h in a vacuum oven at 80 °C. The final electrode has a mass loading of ∼5 mg, as determined for every sample by weighting the nickel plate before coating and the nickel plate plus coating individually.

### Electrochemical Measurements

The as-prepared LiGaO_2_ electrodes were tested as positive electrodes against Li metal using a glass fiber separator (Whatman GF/D) and a polypropylene separator saturated with 1 M LiPF_6_ in an ethylene carbonate and dimethyl carbonate (vol 1:1) electrolyte (LP30, battery grade, Sigma-Aldrich). The cells were assembled in Swagelok cells. Each cycling protocol started with a 20 h open circuit voltage (OCV). Afterward, cyclic voltammetry (CV) and galvanostatic charge/discharge experiments were carried out. The CV was recorded with a scanning rate of 0.02 mV s^–1^. Three different potential ranges are applied in the CV: 0.6–4.2 V vs Li/Li^+^, 0.05–3 V vs Li/Li^+^, and 0.05–4.5 V vs Li/Li^+^. At the upper and lower cutoff potentials, the potential was held for 20 h each. The same CV protocol was applied to initialize and monitor each cell for one cycle before the cells were measured through galvanostatic charge–discharge. The galvanostatic charge/discharge tests were performed at a current density of 10 mA g^–1^ in a potential range from 0.05 to 4.5 V vs Li/Li^+^. At the respective cutoff potential, the potential was held for another 20 h for constant potential charge and discharge, respectively. To monitor the cell’s equilibrium state afterwards, OCV was applied for 20 h before the next charge or discharge started. At least 7 cycles were recorded.

To prepare samples for the *ex situ* measurements, battery cells are assembled as described above and brought to the desired potential within the first CV cycle at a scanning rate of 0.02 mV/s. At each desired potential, a constant potential period of 20 h was applied, followed by an OCV period until the potential equilibrated. Table 1 gives an overview of the investigated *ex situ* samples: A-1 (for the OCV potential after equilibration of the just assembled cell), B-1 (4.5 V vs Li/Li^+^), C-1 (0.05 V vs Li/Li^+^), and D-1 (2.75 V vs Li/Li). Furthermore, *ex situ* samples were taken in the last (7th) cycle of the charge–discharge measurements at the upper and lower cutoff potential labeled as B-7 (4.5 V vs Li/Li^+^) and C-7 (0.05 V vs Li/Li^+^).

**Table 1:** Overview of the Investigated *Ex Situ* Points A-1, B-1, C-1, D-1 from the 1st CV Cycle, and B-7, C-7 from the 7th Cycle via Charge–Discharge

*ex situ* sample	condition	*ex situ* sample	condition
A-1	OCV before 1st cycle 2.75 V vs Li/Li		
B-1	after 1st charge to 4.5 V vs Li/Li^+^	B-7	after 7th charge to 4.5 V vs Li/Li^+^
C-1	after 1st discharge to 0.05 V vs Li/Li^+^	C-7	after 7th discharge to 0.05 V vs Li/Li^+^
D-1	1st cycle complete 2.75 V vs Li/Li		

### X-Ray Powder Diffraction

X-Ray powder diffraction (XRPD) was used to measure the LiGaO_2_ powder after synthesis and the cycled *ex situ* samples (A-1, B-1, C-1, D-1, B-7, and C-7). The LiGaO_2_ powder sample was prepared by using the “front-load” method. A lab diffractometer (Empyrean, Panalytical) with a copper anode (Kα wavelength: 1.54 Å) in reflection geometry was used to measure the LiGaO_2_ pristine powder from 10 to 120°2θ, with a counting time of 30 s per step and a step size of 0.008°2θ. The *ex situ* samples were taken from the disassembled cells in a glovebox, mounted on Si substrates with Scotch Magic tape, and placed in a sample holder. The tape can be well penetrated by X-rays but protects the sample from direct contact with the atmosphere during the measurement.^25^ A lab diffractometer (D8 Discover, Bruker) with a copper anode (Kα wavelength: 1.54 Å) in reflection geometry was used to measure the *ex situ* samples in an angular range from 10 to 120°2θ, with a counting time of 20 s per step and a step size of 0.02°2θ. Structural analysis and quantitative phase analysis (QPA) based on the XRPD data were carried out within the software package Diffrac.Topas (Bruker). The LiGaO_2_ powder sample was structurally analyzed according to the Rietveld method.^26^ The *ex situ* samples were analyzed by QPA from a full pattern fit of the XRPD data.^27^

### Micro-Raman Spectroscopy

Micro-Raman spectroscopy was carried out with a WITEC alpha300R microscope using a solid-state 532 nm excitation laser and 600 L/mm grating with a laser power of 10 mW. The Raman spectra were collected with a 100× objective on an area of 80 μm × 80 μm and a 0.5 μm step raster for the pristine and *ex situ* samples of the first cycle. This yields 160 × 160 pixels, each containing an individual Raman spectrum. Due to the very low Raman activities and huge topography of the *ex situ* samples in the seventh cycle, the Raman analysis was carried out via volume mapping (by changing the stage height, i.e., focus point) in a volume of 50 μm × 50 μm × 50 μm with a 1 μm step size, yielding 50 × 50 × 50 pixels containing each one individual Raman spectrum. Each individual measurement at a point (pixel) was carried out with a 1 s acquisition time for both the volume and area mappings. The collected scans were treated by correction algorithms for cosmic ray removal, noise filtering, and baseline correction. Afterward, the data sets were analyzed through principal component analysis within the WITEC software package “WITEC project”.

### Scanning and Electron Microscopy and Energy-Dispersive X-Ray Spectroscopy

Both, top views (A-1, B-1, and C-1 and B-7) and cross-section analyses (B-7) were carried out to investigate the microstructure and elemental composition via scanning and electron microscopy (SEM) and energy-dispersive X-ray spectroscopy (EDX). The samples were consistently managed under vacuum or argon, and the Swagelok cell disassembly took place within an argon atmosphere inside a glovebox (MBraun and GS).

For top-view measurements, samples were investigated without further treatment, with the sample attached onto the SEM sample holder by carbon tape. For the cross-section preparation, the samples, including the Ni current collector, were divided into quarters using a Buehler IsoMet low-speed saw operating at a rotational speed of approximately 30 rpm. Subsequently, one of the quarters was ground using 3 M diamond sandpaper on a JEOL. A handy-lap grinder, starting with a grain size of 15 μm, progressing through 9, 3, 1, and 0.5 μm. Following this process, the ground surface underwent cross-sectional polishing in a JEOL IB-19530 CCP, utilizing an accelerating voltage of 4 kV. Active LN2 cooling to −100 °C was applied to prevent the temperature elevation of the sample. During the cross-sectional polishing, approximately 100 μm of the material was removed. The cross-section sample was affixed onto an SEM sample holder at a 90° angle, ensuring the polished side faced upward.

SEM images were collected on a Quanta FEG 650 FEI, USA, using an accelerating voltage of 5–20.0 kV with a secondary electron detector. Additionally, an FEI Helios NanoLab 460F1 FIB-SEM was used for a concentric back-scattered (CBS) image for the cross-sectional sample. For the EDX-analysis, an Ametek Octane Super at an accelerating voltage of 20 kV was employed on the top-view sample and an Ametek Octane Elite Super at an accelerating voltage of 5 kV for the cross-sectional sample.

### X-Ray Photoelectron Spectroscopy and Electron Energy Loss Spectroscopy

Synchrotronic XPS measurement for LiGaO_2_ at seventh cycle (charged state) and metallic Ga (as a reference) were performed in a 14A beamline of the Taiwan light source. Cryogenic electron energy loss spectroscopy (Cryo-EELS) measurements were conducted using an FEI Titan G2 ChemiSTEM 80–200 transmission electron microscope (TEM) equipped with a high-brightness field emission gun and probe spherical aberration (Cs) correction system operated at 200 kV. To mitigate electron beam damage, the sample was characterized by using a cryo-transfer holder (Simple Origin, Model 200). The EEL spectra were acquired in scanning transmission electron microscopy (STEM) mode with an annular dark-field (ADF) detector. The convergence semiangle was ∼25 mrad.

## Results and Discussion

### Structural Characterization of LiGaO_2_ Powder

**Figure 1:**
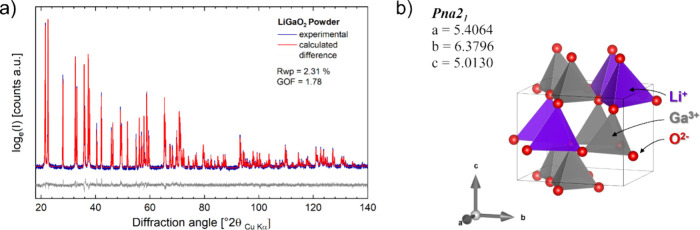
(a) Results of Rietveld refinement for XRPD of LiGaO_2_: experimental XRPD data (blue), calculated data (red), and difference of *I*_obs_ – *I*_calc_ (gray). Whole fitting range (17–140° 2θ) presented. (b) Visualization of the unit cell of LiGaO_2_ in spacegroup *Pna*2_1_.

Figure 1 presents the X-ray powder diffraction pattern of the synthesized LiGaO_2_. The observed Bragg peaks can be assigned to a reference structure of β-LiGaO_2_ in space group *Pna*2_1_ (ICSD coll. code 18152).^20^ This phase was also used as a starting model in the following Rietveld analysis. The fit was carried out considering the LiGaO_2_ scale factor, sample displacement, size, and strain broadening and through refining all relevant structural parameters, such as lattice parameters, Debye–Waller factors, fractional coordinates, and occupancies. All structural parameters (except Li fractional coordinates and Li isotropic Debye–Waller factors) were freed to converge. Their final results are given in Table 2. The lattice parameters only slightly deviate from the model structure (deviation less than 0.1%), and fractional coordinates only slightly shift compared to the model structure (shift less than 2%). There are no secondary phases visible in the pattern. Hence, our synthesis produced phase pure β-LiGaO_2_ via solid-state reaction at 1200 °C in the space group *Pna*2_1_. We are thus reporting the identical compound of LiGaO_2_ in space group *Pna*2_1_, which has been reported in the literature and has been extensively studied for its crystal structure,^20,28^ band structure,^29,30^ application as a substrate for GaN thin film deposition,^18,19,31^ and employment as a dielectric ceramic.^32^

**Table 2:** Refined Structural Parameters in Rietveld Refinement against Measured XRPD Data for the LiGaO_2_ Powder Sample (see Figure 1)

LiGaO_2_; *Pna*2_1_; *a* = 5.406400(17), *b* = 6.37962(2), *c* = 5.013078(15); Rwp = 2.31%; GOF = 1.78						
Wyckoff position	atom type	fractional coordinate *x*	fractional coordinate *y*	fractional coordinate *z*	site occupancy	isotropic Debye–Waller factor (*B*_iso_) [nm^2^]
*4a*	Li	0.42070	0.1267	0.49360	1	0.612
*4a*	Ga	0.08261(9)	0.12541(8)	0.0	1	0.125(11)
*4a*	O	0.3974(4)	0.1430(8)	0.8876(3)	1	0.50(4)
*4a*	O	0.0628(4)	0.1105(9)	0.3633(3)	1	0.50(4)

Furthermore, the synthesized LiGaO_2_ polymorph is expected to be identical to the LiGaO_2_ secondary phases in the Ga:LLZ system that have been reported in the literature.^7,9,10^ It is known that, besides the polymorph in spacegroup *Pna*2_1_, three other polymorphs exist for LiGaO_2_. These polymorphs crystallize in either the α-NaFeO_2_ structure type (spacegroup *R*3̅*m*),^33^ the NaCl structure type (spacegroup *Fm*3̅*m*),^22^ or in the β-LiFeO_2_ structure type (spacegroup *I*4/*m*).^22^ In the literature, the LiGaO_2_ secondary phases were mostly identified from the main characteristic feature at 21–23°2θ in XRPD. Only LiGaO_2_ in spacegroup *Pna*2_1_ possesses this characteristic feature, due to its two main reflections 1 1 0 and 0 1 1 at 21.66 and 22.38°2θ, respectively. The other reported polymorphs are either high-pressure phases or metastable and thus unlikely to be formed during the reaction of Ga:LLZ at high temperature (>950 °C) and ambient pressure.^7,9,10^ Thus, we expect that the herein reported LiGaO_2_ polymorph in space group *Pna*2_1_ is the very same polymorph that has frequently been reported to coexist as a secondary phase with Ga:LLZ garnet.^7,9,10^

### Electrochemical Analysis of LiGaO_2_ Electrodes

**Figure 2:**
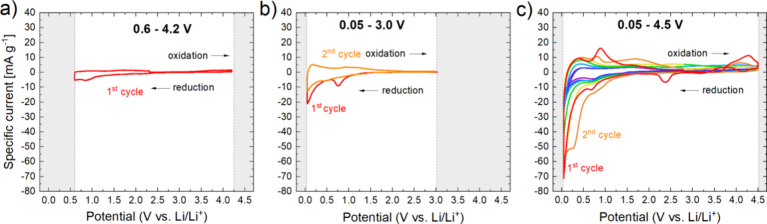
Cyclic voltammetry of LiGaO_2_ against Li metal in an electrochemical test cell for (a) potential window 0.6–4.2 V vs Li/Li^+^; (b) potential window 0.05–3.0 V vs Li/Li^+^; and (c) potential window 0.05–4.5 V vs Li/Li^+^.

The as-synthesized LiGaO_2_ powder was processed into electrodes by a typical procedure to be tested against Li metal in a laboratory cell.^34,35^ Prior to each CV scan, the cells were set to the OCV for 20 h to reach their equilibrium potential between 2.3 and 2.5 V vs Li/Li^+^. Figure 2 displays the results of the electrochemical activities during the CV scans in different electrochemical potential windows. In the first tested electrochemical potential window (Figure 2a), from 0.6 to 4.2 V vs Li/Li^+^, a small nonreversible reduction reaction was observed at ∼0.8 V vs Li/Li^+^. In the second tested electrochemical potential window (Figure 2b), from 0.05 to 3.0 V vs Li/Li^+^, the CV scan discloses a more significant activity in the lower electrochemical potential region below 0.6 V vs Li/Li^+^, peaking −20 mA g^–1^ upon reduction at 0.05 V vs Li/Li^+^_._ The reaction shows reversibility but degrades quickly. The third tested electrochemical potential window is also the widest window tested, reaching from 0.05 to 4.5 V vs Li/Li^+^ (Figure 2c). The CV records additional activities above 3.0 V vs Li/Li^+^ upon oxidation and a dramatically increased activity below 1 V vs Li/Li^+^, peaking at −70 mA g^–1^ in the first cycle upon reduction. The reactions display reversibility, although incomplete, as can be seen from the absence of symmetries in the oxidation and reduction curves. A large degradation of activity is observed upon cycling, leading to a fade of the peak current at 0.05 V vs Li/Li^+^ during reduction to less than −10 mA g^–1^ in the seventh cycle.

The results demonstrate that the activity of LiGaO_2_ strongly depends on the choice of the lower and upper cutoff potential. The recorded activities above 3 V vs Li/Li^+^ may be critical for the access of the full activity in the low potential region, as can be concluded from the comparison of cycling to 3.0 and 4.5 V (Figure 2b,c). The displayed behavior in the CV gives a strong hint that a combination of high-voltage decomposition (or activation) and subsequent reversible reactions lead to a significant electrochemical activity through LiGaO_2_ decomposition products in the potential window from 0.05 to 1.0 V vs Li/Li^+^.

**Figure 3:**
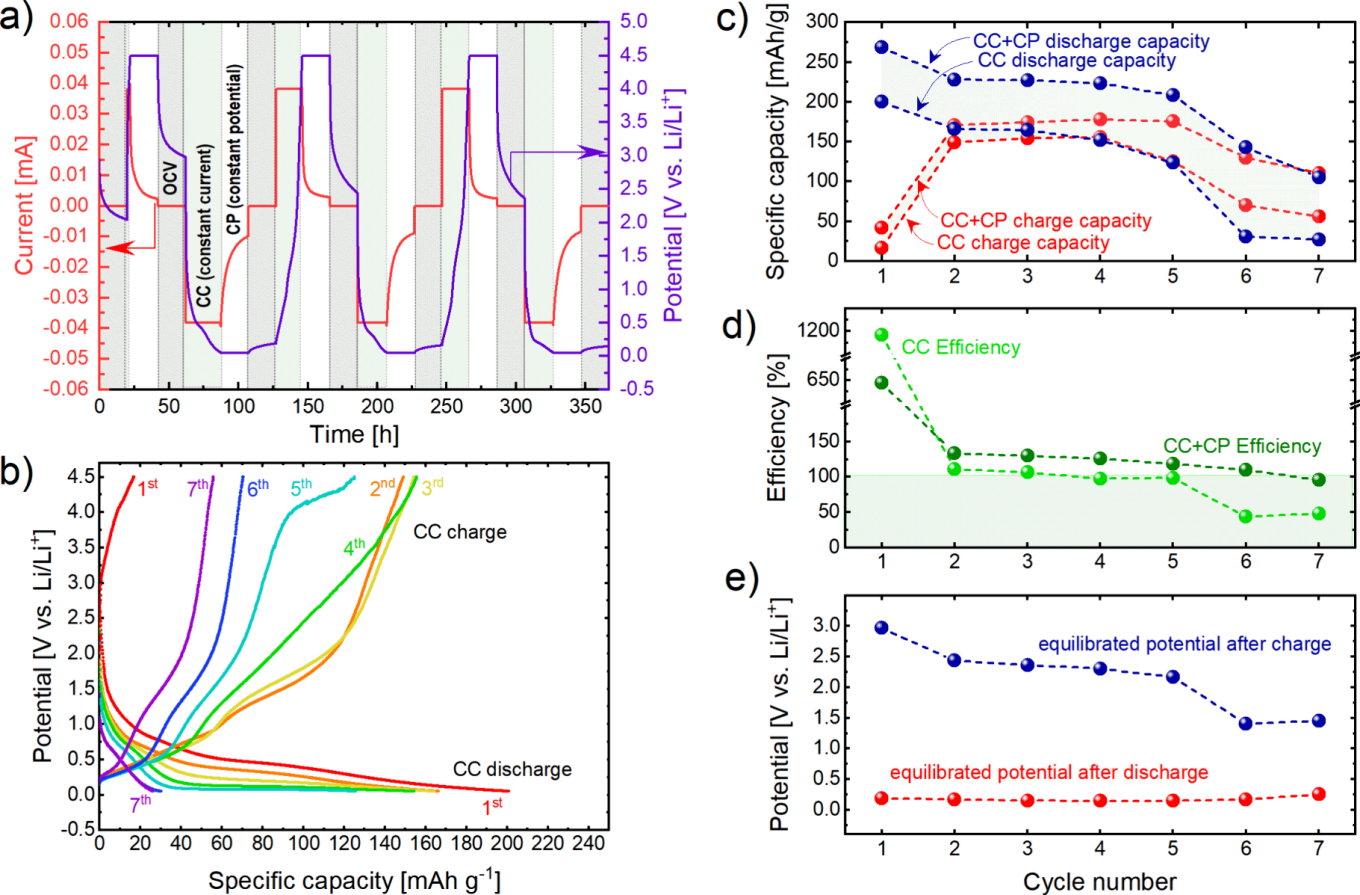
Results of the constant current (CC) + constant potential (CP) charge and discharge of LiGaO_2_ against Li metal in an electrochemical test cell. (a) Cycling protocol with indicated OCV, CC, and CP periods; (b) potentials over capacities achieved with CC charge/discharge for the 1st to the 7th cycle; (c) achieved CC capacities in comparison to achieved CC + CP capacities as a function of cycle number; (d) efficiency as discharge capacity divided by charge capacity for CC and CC+CP as a function of cycle number; and (e) potential after each 20 h OCV period (“equilibrated” potential) as a function of cycle number.

To investigate the electrochemical behavior deeper, a dedicated charge–discharge cycling protocol was applied to freshly prepared cells. The cycling protocol applied an initial OCV and 1 CV cycle followed by constant current (CC) and constant potential (CP) charge and discharge in the potential range from 0.05 to 4.5 V vs Li/Li^+^ (Figure 3a). After each CC and CP dis/charge, an OCV of 20 h was applied to allow for the potential equilibration. Figure 3b demonstrates the achieved CC capacities and voltage profiles over 7 cycles. Like the CV results, the reaction activity is mostly concentrated at lower potentials <1.5 V vs Li/Li^+^. Also, a reversible reaction is demonstrated over several cycles, with significant degradation of the achieved CC capacities.

Figure 3c displays the achieved CC charge and discharge capacities over cycle number and compares them with the achieved CC+CP discharge capacities over cycle number. It is obvious that applying a CP step after the CC steps yields 25–55 mAh g^–1^ additional charge capacity and 70–115 mAh g^–1^ additional discharge capacities. In the first cycle, a CC+CP charge capacity of 42 mAh g^–1^ is recorded, followed by a discharge capacity of 269 mAh g^–1^ and a second charge capacity of 172 mAh g^–1^. In the seventh cycle, 109 and 106 mAh g^–1^ are recorded upon charge and discharge, respectively. In general, the CP charge and discharge capacities tend to get larger with a higher cycle number, while the CC+CP charge capacities first rise and then fade, whereas the discharge capacities show a steady fade. The corresponding efficiencies as a function of the cycle number are displayed in Figure 3d. Very large CC and CC+CP efficiencies are recorded for the first cycle: the discharge capacities are 1195% (CC) and 640% (CC+CP) higher than the charge capacities in the first cycle. In the second cycle, they fall to 111% (CC) and 136% (CC+CP) and fade slowly to 51% (CC) and 97% (CC+CP) in the seventh cycle.

If all Li can be extracted from LiGaO_2_ during charging, then it would have a theoretical capacity of 247 mAh g^–1^. The first charge capacity (42 mAh g^–1^) thus suggests that up to 17% of the total amount of Li in the LiGaO_2_ electrode has been extracted and transported to the negative electrode upon releasing one electron per extracted Li into the outer circuit during the CC+CP charge. As indicated by the high discharge capacity of the first cycle (269 mAh g^–1^), 640% more Li is brought back into the positive electrode during the following discharge than was taken out during charge. This is only possible if a reaction product with a high Li storage capacity has formed upon charging in the first cycle. In the further cycles, this compound may undergo reversible electrochemical reactions with Li, as can be concluded from the reversible nature of the displayed activities in CV and galvanostatic charge–discharge cycling. Furthermore, the decomposition of LiGaO_2_ into materials that allow to reversibly react to high amount of Li seems to be ongoing, at least until the sixth cycle, because of the recorded efficiencies above 100%.

During each OCV period between CC+CP charge and discharge, the evolution of the cell potential was recorded for20 h. As can be seen in Figure 3a, there is a significant potential drop in each OCV period after charging to 4.5 V vs Li/Li^+^ and a noticeable potential rise during each OCV period after discharging to 0.05 V vs Li/Li^+^. After the first charge, the potential falls from 4.5 to 2.9 V vs Li/Li^+^ during 20 h of the OCV period. The potential after this 20 h OCV does not reach the equilibrium potential yet, as an asymptotic behavior of the potential-overtime function is not yet displayed. After the first discharge, the potential value rises from 0.05 to 0.2 V vs Li/Li^+^ during 20 h of OCV. Here, an asymptotic behavior is displayed, and the cell seems to be in equilibrium. Figure 3e displays the (pseudo) equilibrated potential values at the end of each OCV period. As can be seen, the potentials after discharge rise slightly after the individual cycle, from 0.20 to 0.24 V vs Li/Li^+^. On the other hand, the recorded potentials at the end of a 20 h OCV after charge show a continuous decrease over cycle number from 2.9 V vs Li/Li^+^ to 1.45 V vs Li/Li^+^.

The potential equilibrations to higher and lower potentials during an OCV period after charge and discharge, respectively, are typical for electrochemical cells due to polarizations and diffusion limitations. The potential will naturally equilibrate with the specific electrode potential of the material at the specific state of charge. Slower reaction kinetics during charge or discharge will usually lead to a stronger potential drop during OCV. The potential rise from 0.05 to 0.2 V vs Li/Li^+^ in the OCV period after the first discharging thus suggests that the reaction has not been completed and a compound with an electrochemical potential of 0.2 V vs Li/Li^+^ is formed. Most likely, the reaction would have kept going, if a smaller discharge current and a longer CP period had been applied. This is also supported by the observed decrease of the current during the CP period, which does not show full completion of the asymptotic trend yet after 20 h. This means the compound that we observe at the end of discharge is only partially electrochemically reacted, which is important information for interpretation of the later *ex situ* experiments. Furthermore, the slight rise in electrochemical potential during the individual OCV periods after each discharge suggests that the reaction kinetics worsen during cycling, meaning that compounds with different discharge states are produced at the end of each discharge cycle.

The potential drop in the OCV period after charging is significant and implies an additional underlying mechanism in addition to limited reaction kinetics. After the first charge, the potential during OCV drops back to a value the same as the pristine cell (2.95 V vs Li/Li^+^), even though a significant capacity had been recorded. The dominant mechanism here seems to be the decomposition of LiGaO_2_ above 4 V vs Li/Li^+^. Obviously, the decomposition product must have a lower electrochemical potential than LiGaO_2_, which is why the cell tends to equilibrate back to the electrochemical potential of LiGaO_2_ during the OCV periods after discharge (which has the highest electrochemical potential in the formed composite material). Interestingly, the recorded potentials at the end of each OCV period decrease to 2.1 V vs Li/Li^+^ in the fifth cycle and then drop to 1.41 and 1.45 V vs Li/Li^+^ in the sixth and seventh cycles. The significant drop of the level of OCV means that LiGaO_2_ must have either completely reacted into a different compound or has been isolated within the electrochemical cell in the sixth and seventh cycles, i.e., through microstructural degradation and loss of contact between LiGaO_2_ and the reactions products, binders, and carbon.

Even though significant capacity degradation was observed, the underlying reversible nature in the following cycles strongly suggests that the formed product is a compound with a high reversible capacity in the lower potential range <1.5 V vs Li/Li^+^. The electrochemical characteristics show striking similarities to the electrochemical reaction of Ga_2_O_3_ vs Li/Li^+^.^36−38^ In these studies, Ga_2_O_3_ is intentionally decomposed to Ga metal to form Li–Ga alloy electrodes.^36−38^

### Structural and Microstructural *Ex Situ* Analyses

**Figure 4:**
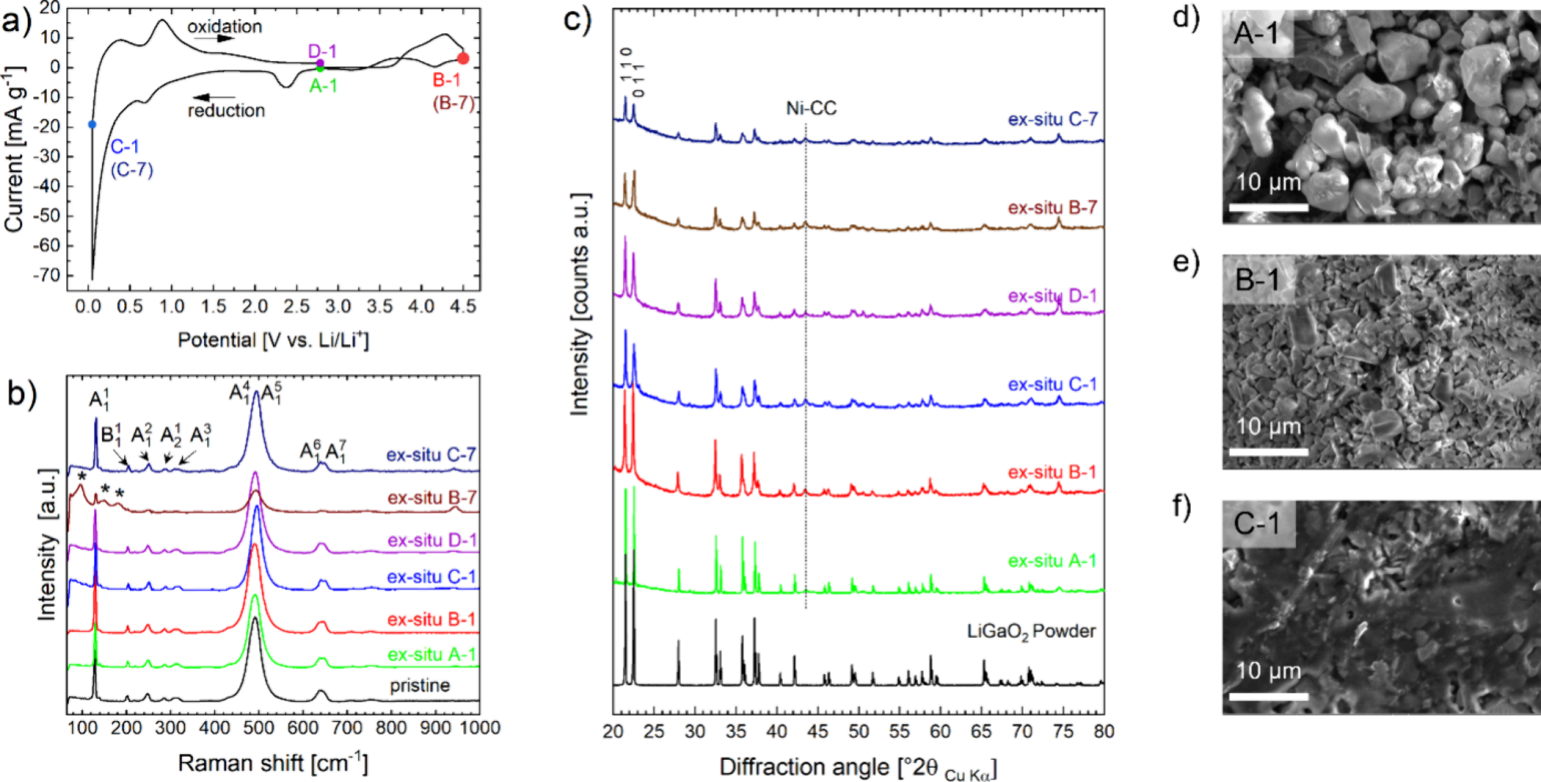
*Ex situ* analyses of the cycled LiGaO_2_ electrode. (a) Indication of investigated *ex situ* points A-1, B-1, C-1, D-1 from the 1st CV cycle, B-7 and C-7 from the 7th cycle via charge–discharge. Reflections of the Ni current collector (Ni-CC) substrate are indicated by a dashed line; (b) averaged micro-Raman spectra from mapping areas of 80 × 80 μm (pristine electrode, A-1, B-1; C-1, D-1) and 50 × 50 μm (B-7, C-7) on current collectors; (c) XRPD data of the LiGaO_2_ powder sample and A-1 to B-7 electrode samples on Ni-CC. (d–f) SEM images through secondary electron detection for A-1 (d), B-1 (e), and C-1 (f).

To further understand the electrochemical reactions of LiGaO_2_, *ex situ* analyses via SEM, XRPD, and micro-Raman spectroscopy were carried out. Figure 4a indicates the measured voltages of the *ex situ* samples, which are also listed in Table 1. A-1 was taken after the initial OCV for potential equilibration, which has an equilibrated potential of 2.0 V vs Li/Li^+^. B-1 was taken after oxidation through CV scan to 4.5 V vs Li/Li^+^ and then set to OCV for 20 h. The final equilibrated potential of the cell was 3.0 V vs Li/Li^+^. C-1 was taken after oxidation to 4.5 V and reduction to 0.05 V vs Li/Li^+^ and resting at 0.05 V vs Li/Li^+^ for 20 h. It has an equilibrated potential of 0.22 V vs Li/Li^+^. D-1 is taken after one full cycle plus rest (oxidation to 4.5 V and reduction to 0.05 V vs Li/Li^+^ and another oxidation to 2.75 V vs Li/Li^+^ and resting at 3.0 V vs Li/Li+ for 20 h). It has an equilibrated potential of 2.5 V vs Li/Li^+^. B-7 is taken at the position of B-1 but after 7 additional cycles of charge–discharge. It has an equilibrated potential of 1.5 V vs Li/Li^+^. C-7 is taken at the position of C-1 but after 7 additional cycles of charge–discharge. It has an equilibrated potential of 0.25 V vs Li/Li^+^.

The results from Raman microscopy are shown in Figure 4b. Interestingly, the averaged Raman spectra all show the very same features and not much deviation from each other. As such, the recorded spectra for the pristine sample but also all the *ex situ* samples agree well with the Raman spectrum of β-LiGaO_2_. β-LiGaO_2_ has point group *C*_2*v*_ with experimental confirmed bands at 128.7 cm^–1^ (A_1_^(1)^), 204.2 cm^–1^ (B_1_^(1)^), 252.1 cm^–1^ (A_1_^(2)^), 289 cm^–1^ (A_2_^(1)^), 444.3 cm^–1^ (A_1_^(3)^), 493.3 cm^–1^ (A_1_^(4)^), 502.1 cm^–1^ (A_1_^(5)^), 643.9 cm^–1^ (A_1_^(6)^), and 653.8 cm^–1^ (A_1_^(7)^).^28,39^ Accordingly, the Raman spectra for the pristine sample and *ex situ* A-1, B-1; C-1, D-1, and C-7 were indexed to the bands of β-LiGaO_2_ in point group *C*_2*v*_. Only the spectrum for the B-7 sample cannot be assigned to β-LiGaO_2_ alone. Here, the bands from the β-LiGaO_2_ modes seem to have vanished while new, weak, and broad bands em**e**rged at 99, 145, and 182 cm^–1^ (indicated by * in Figure 4b), which will be discussed in more detail later.

Similarly, the XRPD patterns (Figure 4c) show LiGaO_2_ in *Pna*2_1_, as the main phase in all *ex situ* samples, resembling the pristine powder. There are changes in the intensity ratios of the two main reflections, and shoulders emerge predominantly at the *0 1 1* reflection at 22.6° (Figure S1 in the Supporting Information provides a more detailed view). Further, it is noted that the reflection maximum intensities get weaker, and the Bragg peaks tend to broaden throughout the sample series. The peak broadening suggests a significant change in a microstructure, i.e., crystallite sizes or strains of LiGaO_2_ crystallites. At this stage, it must be assumed that any possible decomposition products are hard to detect through Raman spectroscopy and XRPD where their only evidence may lay in the subtle changes in the spectra (e.g., *ex situ* sample B-7) and their diffraction patterns (e.g., intensity ratios and peak shoulders in B-1 to C-7).

As the Bragg peak broadening is the most obvious from samples A-1 to B-1, electron microscopy was used as a complementary tool for understanding the underlaying mechanism. A drastic change in the microstructure was observed in the SEM images for the sample A-1 to the sample B-1 (Figure 4d,e) where sample A-1 shows LiGaO_2_ particles with a size distribution from 1 to 10 μm, with most of the particles in between 7 and 10 μm and sample B-2 on the other hand shows smaller particle distribution between 1 and 5 μm and only a few particles above 7 μm. These results suggest that, even though LiGaO_2_ seems to be majorly preserved in the *ex situ* samples, it does undergo a significant microstructural change. For sample C-1 (Figure 4f), a new microstructural feature is present, which has the appearance similar to a melt. Similarly, optical images taken for all *ex situ* samples show a drastic changing microstructure from a pure powder-like sample to a solidified specimen under the presence of a melt phase (see Figure S2, Supporting Information).

### Quantitative Phase Analysis from XRPD

The possible presence of Li–Ga alloys or Ga was further analyzed through quantitative phase analysis based on the collected XRPD data. In parallel, the LiGaO_2_ phase was quantified with respect to its lattice parameters and crystallite sizes to understand its contribution to the reactions during cycling. To describe the main LiGaO_2_ phase in the diffractograms, the refined structural parameters of the LiGaO_2_ powder sample (Figure 1, Table 2) were used as the starting values. Parameters like the fractional coordinates, Debye–Waller factors, and site occupancies were kept fixed at their refined values from the LiGaO_2_ powder sample, while lattice parameters, sizes, and strains were refined against the measured data. Besides LiGaO_2_ and Ni (as the CC), possible impurity phases such as Li_3_N, Li_2_O, LiOH, LiOH·H_2_O, and Li_2_CO_3_ and a series of possible reaction products such as Ga_2_O_3_, LiGa_5_O_8_, Li_5_GaO_4_ as well as various Li–Ga alloys, and Ga metal were testes within the quantitative phase analysis routine. It was concluded that none of the above-mentioned phases could be fitted reasonably against the measured data besides Ni, Li_3_N, and some specific compositions of a Li–Ga alloy. Structural information files for these samples were taken from ICSD references as listed in Table S1, Supporting Information.^20,40−43^

**Figure 5:**
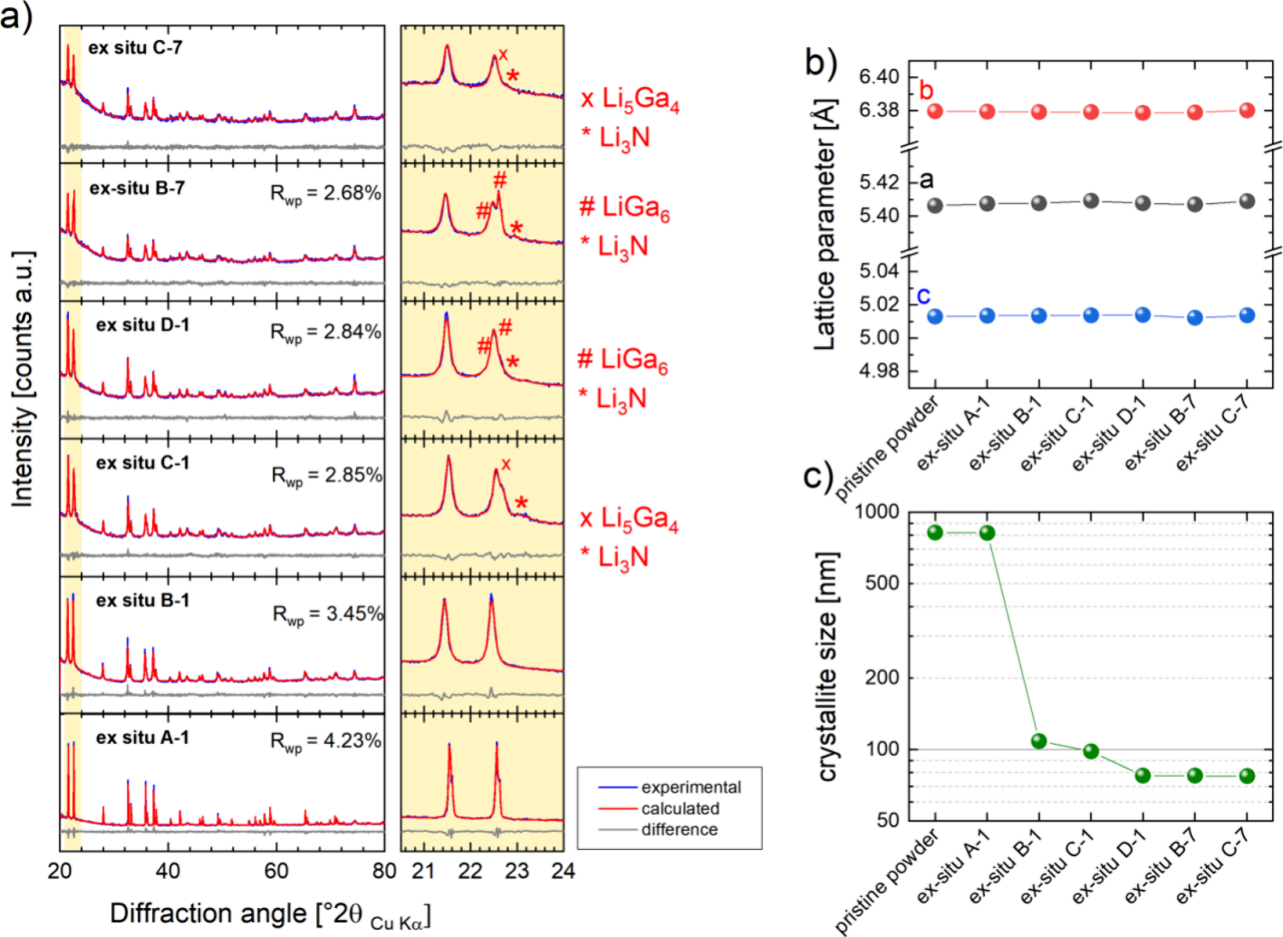
Results of the QPA based on XRPD data of the *ex situ* samples A-1 to C-7 (a) experimental, calculated, and difference for the fits in the fitting ranges 17–80°2θ; (b) evolution of refined LiGaO_2_ lattice parameters over the sample series; and (c) evolution of refined crystallite sizes of the sample series.

Figure 5a shows the fitting results of QPA for the sample series. The evolution of the refined lattice parameters and crystallite sizes is shown in Figure 5b,c. For comparison, the refined lattice parameters and crystallite size for the pristine LiGaO_2_ powder sample are shown, too. As can be seen, the refined values for the lattice parameters remain constant throughout the sample series, while the crystallite sizes show a clear decreasing trend upon cycling. The crystallite size for the *ex situ* A-1 (OCV equilibrated) sample refines to a similar value (820 nm) than the refined crystallite size for the pristine powder sample (823 nm). For the B-1 sample, the crystallite size refined significantly smaller to 108 nm. Over the sample series, this value steadily decreases and refined to 77 nm for C-7.

The XRD patterns for C-1, D-1, B-7 and C-7 cannot be described from the contribution of a LiGaO_2_ phase alone. A small fraction of Li_3_N contaminations must be considered, which most likely arises from reactions with the ambient atmosphere. Even though the sample was protected from the atmosphere by the Scotch magic tape, these reactions can happen if highly reactive phases (such as Li or Li–Ga alloys) are present in the sample. Neither Ga_2_O_3_ nor Li–Ga–O related phases, such as LiGa_5_O_8_ or Li_5_GaO_4_ could be fitted against the measured data. Still, C-1, D-1, B-7, and C-7 remained poorly described by considering only LiGaO_2_ and Li_3_N. This is especially evident from the region between 21 and 22°2θ, where shoulders and peaks next to the main reflection of LiGaO_2_ emerge (see Figure 5a, enlarged region).

For *ex situ* C-1 and C-7, the main reflection of Li_5_Ga agrees with the position of the shoulder at 22.5°2θ. However, considering its contribution to the diffraction pattern of the C-1 sample, is successful only if a high texture for its *0 0 l* reflection is considered. Similarly, LiGa_6_ can be fitted against the measured data for D-1 and B-7 and successfully describe the shoulders and peaks and 22.0–22.5°2θ, if a strong texture for its *0 0 l* reflections is considered. While this may indicate the presence of Li–Ga alloys in the sample, it cannot completely exclude the presence of Ga, Ga_2_O_3_, or Li–Ga–O ternary compounds in the samples. Ga metal, which has a melting point at 29.77 °C, is most likely in the liquid state or a supercooled liquid and thus X-ray amorphous. On the other hand, Li–Ga-oxides or Ga_2_O_3_, if decomposed from LiGaO_2_ at higher voltage, are unlikely to show a good crystallinity. They may present as a low-crystalline or nanoscale secondary phase that cannot readily be identified in the given diffraction patterns.

### Component Analyses from Micro-Raman Spectroscopy Mapping

**Figure 6:**
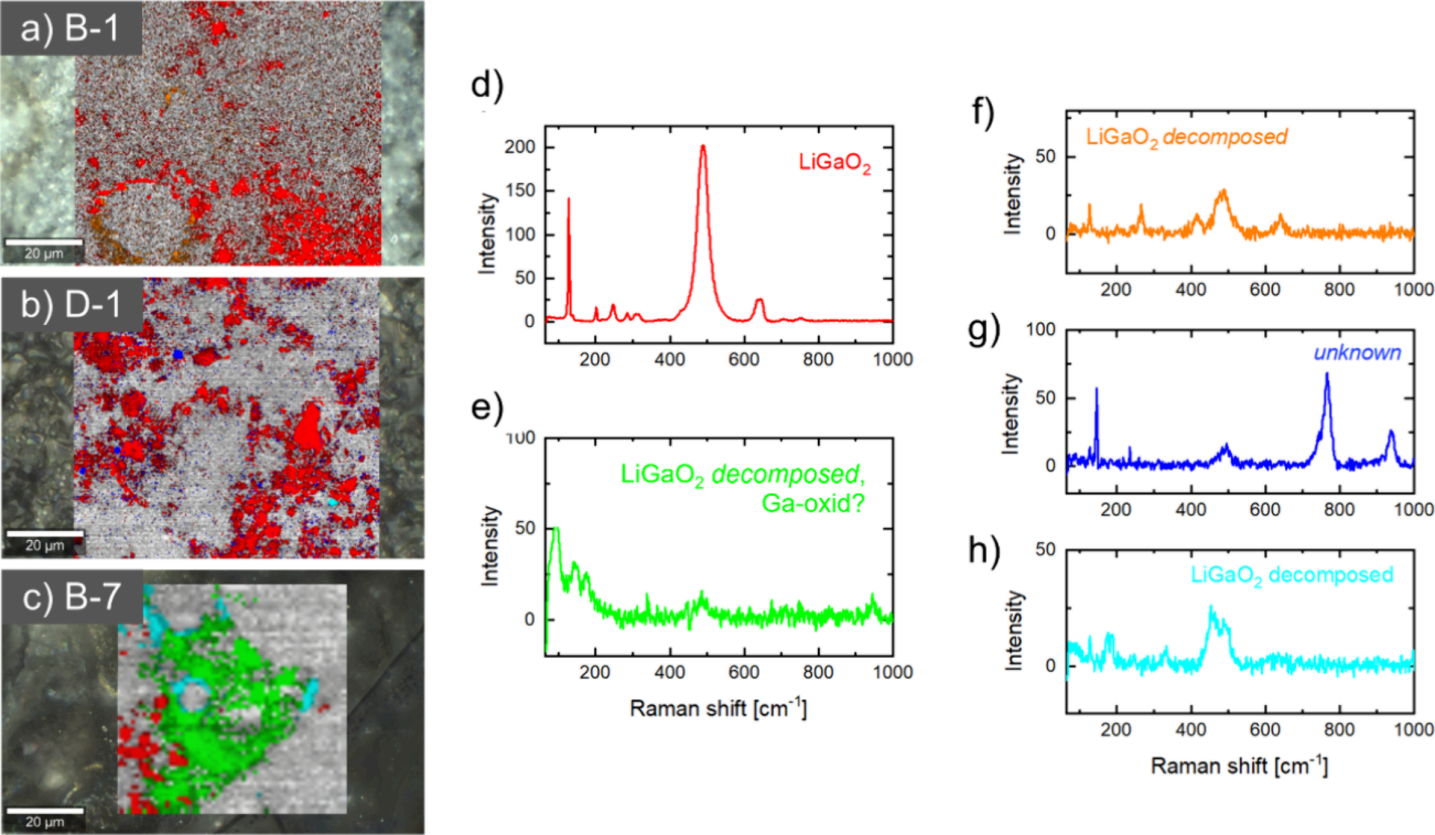
Results of principal component analysis from micro-Raman mappings for the samples (a) B-1 (first charge to 4.5 V vs Li/Li^+^), (b) D-1 (first full cycle), and (c) B-7 after charging to 4.5 V vs Li/Li^+^ in the 7th cycle. Each color represents an identified individual component through the principal component algorithm. Gray areas are out of focus or have intensities that are too low. For each component, a representative individual spectrum is shown: (d) LiGaO_2_, (e) decomposed LiGaO_2_ or Ga-oxide, (f) low intensity area/decomposed LiGaO_2_, (g) unassigned, and (h) low intensity area/decomposed LiGaO_2_.

To investigate possible reaction products in the system further, the collected micro-Raman maps for the samples B-1 (1st charge), D-1 (1st full cycle), and B-7 (7th charge) were analyzed through a principal component algorithm within the WITEC project software package. The results of the analysis are displayed in the color-coded maps in Figure 6a–c, and their representative individual spectra of the identified components are shown in Figure 6d–h. After the first charge and after the first full cycle, the main component in the mapped area was LiGaO_2_ (Figure 6d), as was already represented by the averaged spectrum from the mapped area of the sample B-1 and D-1 in Figure 4b.

In the mapped area of the seventh charge to 4.5 V vs Li/Li^+^, a new main component (Figure 6e) appears to be widely distributed over the mapped area. The component shares the feature of the averaged spectra of sample B-7 in Figure 4b, with a main, yet weak, band at 99 cm^–1^ and 2 more weak bands at 145 and 182 cm^–1^. Compared to LiGaO_2_, the newly identified component has a much lower Raman activity, as can be seen from the absolute intensity scales in Figure 6d,e. From the electrochemical analysis, where similarities with the Ga_2_O_3_ and Ga electrochemical activities vs Li were recorded, decomposition products such as gallium oxides or Ga metal may be expected. Gallium-oxide in the form of Ga_2_O_3_ is known to have many polymorphs each having their own individual Raman spectra.^44−47^ Most of the experimentally recorded Raman spectra for Ga_2_O_3_ do not agree to the observed features for the identified component in Figure 6e. Similarly, not a single band matches the Ga-metal Raman spectrum.^48^ The only similarities are identified for the κ/ε-Ga_2_O_3_ spectrum.^47^ However, due to the low Raman activity and poor signal/noise, the assignment of bands is rather difficult. Besides, in the spectrum, we note that some features of the LiGaO_2_ spectrum are still well preserved, like the A_1_^(4)^ and A_1_^(5)^ modes as the main feature in the LiGaO_2_ spectrum at ∼490 cm^–1^. Furthermore, LiGaO_2_ components with their characteristic spectra have been identified in some areas of the sample dominantly, as shown by the red areas in the map of sample B-7. Three secondary components with a small distribution in the mapped area and a rather weak intensity compared to that of the collected LiGaO_2_ spectrum were identified (Figure 6f–h). The results suggest that, even at the scale of micro-Raman spectroscopy (lateral resolution of 500 nm), single phases cannot be identified. Most likely, the sample is heavily inhomogeneous even at the upper nanoscale. Yet, the presence of Li–Ga alloys or liquid Ga cannot be excluded from being present in the sample because of their Raman inactivity.

### Scanning Electron Microscopy and Energy-Dispersive X-Ray Spectroscopy

**Figure 7:**
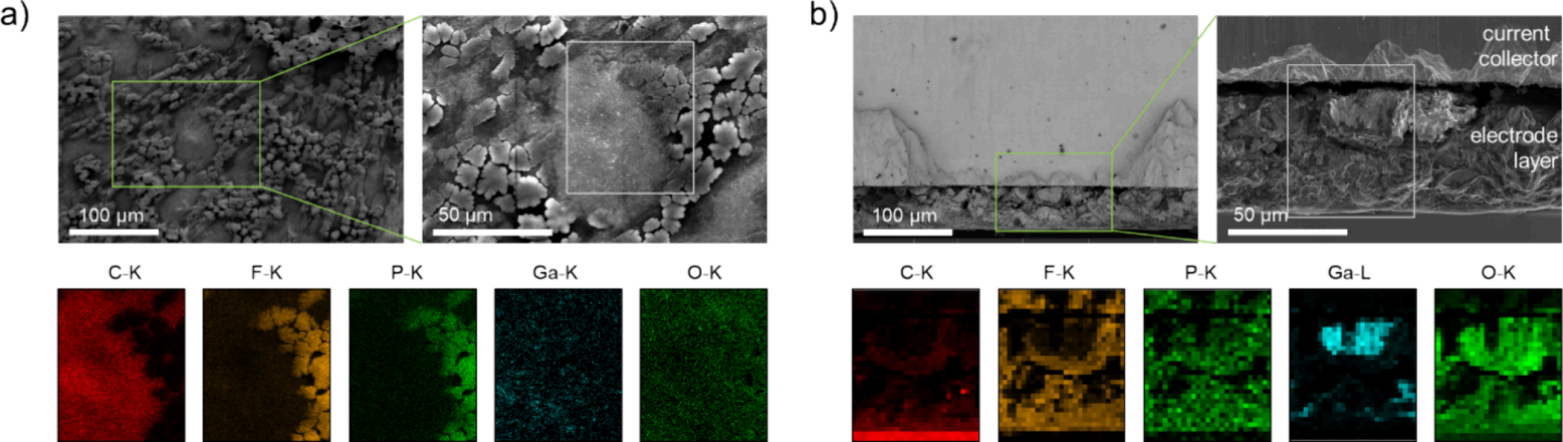
SEM and correlative EDX mappings for (a) top view of the B-7 sample (left accelerating voltage 5 kV, right accelerating voltage 20 kV) and (b) cross section of the B-7 sample (accelerating voltage 5 kV).

As a further step to identify the nature of the possible decomposition and reaction products, SEM and correlative EDX mappings were collected for the B-7 sample (Figure 7). In the top view of the sample, the binder in the electrode and carbon additive can still be clearly identified from its F–K and P–K signals. Carbon seems to be majorly present in the selected area, as evidenced from its C–K signal. However, Ga or O failed to be localized and showed only a weak and broad distribution throughout the investigated area.

The cross-section EDX map reveals a high concentration of C at the surface of the electrode sheet. F–K and P–K signals can be found throughout the electrode layer and indicate the presence of the used binder. Toward the current collector, the microstructure turns from a densified top layer into an agglomeration-like structure with obvious pores and microstructural disintegration of material from the rest of the layer. Yet, the gap between the layer and current collector may be an artifact from the cross-section polishing procedure. Ga can be localized in densified agglomerates, in addition to O and surrounded by an O-rich periphery, as evidenced by the Ga–L and O–K signals (Figure S3, Supporting Information, provides further EDX maps showing similar features on a different area of the sample). Interestingly, the areas where binder is present (F and P rich) show significant signals for the O–K, suggesting that the binder became heavily oxidized or new oxide products have been formed among the matrix.

### X-Ray Photoelectron Spectroscopy and Electron Energy Loss Spectroscopy

**Figure 8:**
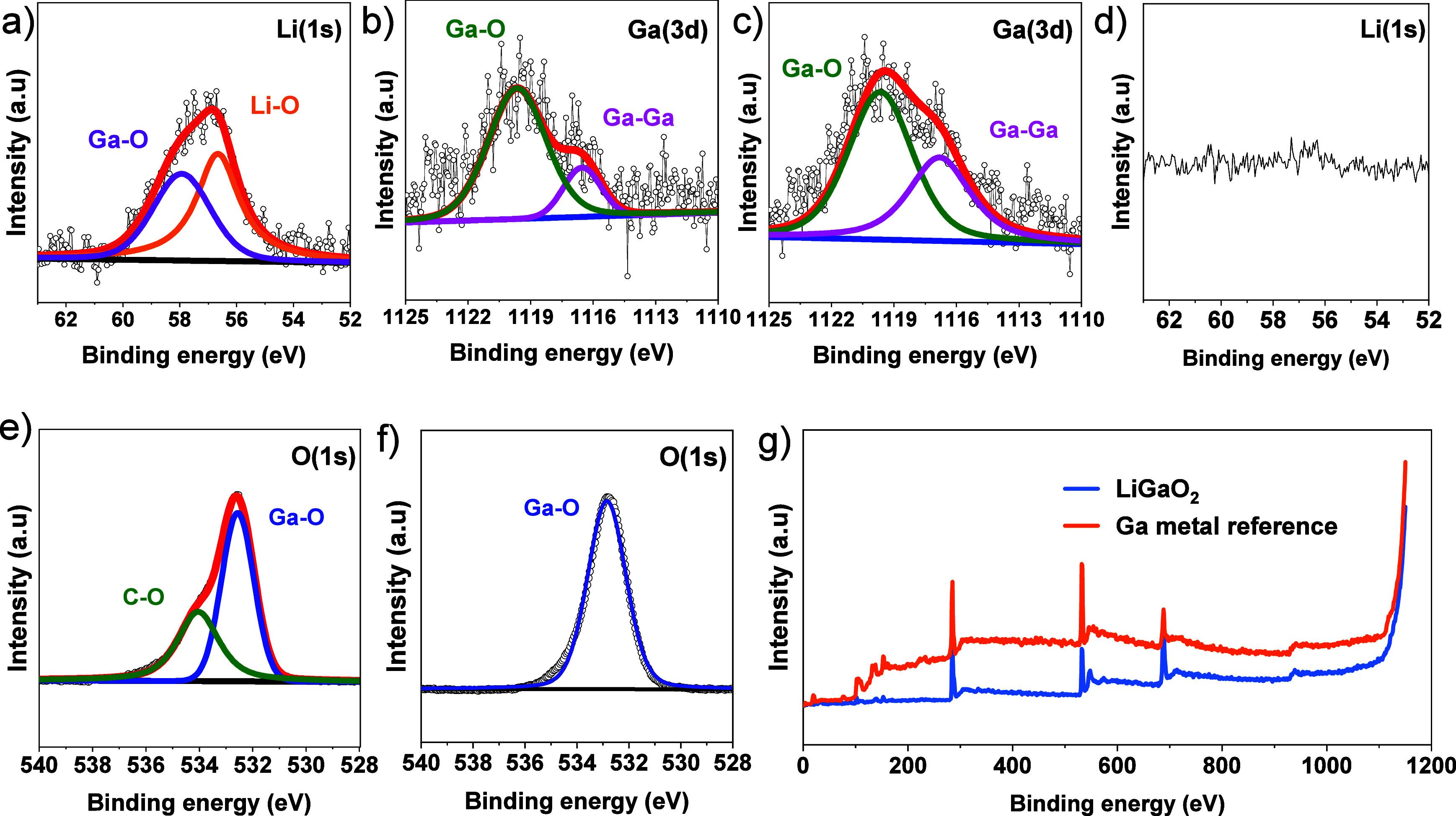
XPS spectra for the B-7 sample and metallic Ga reference sample. Panels a and b are Li(1s) and Ga(3d) spectra for the B-7 sample; panels c and d are Ga(3d) and Li(1s) spectra for metallic Ga samples. Panels e and f are spectra belong to O(2s) for B-7 and metallic Ga, respectively. (g) XPS survey for both B-7 and metallic Ga samples.

Synchrotronic XPS measurement for LiGaO_2_ at seventh cycle (charged state, B-7) and metallic Ga (as a reference) were performed in the 14A beamline of the Taiwan light source. For the LiGaO_2_ sample (B-7), Li(1s) spectra indicate the presence of Li_2_O and LiGaO_2_ from Li–O at around 57 eV and possible Ga_2_O_3_/LiGaO_2_ from Ga–O at 58.27 eV^49−51^ as indicated in Figure 8a–c. However, in Figure 8d, there is no peak belonging to metallic Ga in Li 1s spectra, which was used as a reference. It is interesting to observe the peak ratio difference of Ga–O (1119.65 eV) to the metallic gallium (Ga–Ga) peak (1116.53 eV).^52,59^ The Ga–Ga peak is comparably smaller in the B-7 sample than in the Ga reference; however, it indicates the presence of metallic gallium for the LiGaO_2_ electrode in the charged state after the seventh cycle, which will be further discussed later.

Furthermore, the O(1s) spectra from Figure 8e for the LiGaO_2_ sample shown two peaks associated with C–O (534.06 eV)^5^ from the conductive carbon or electrolyte and Ga–O (532.56 eV) peak from the LiGaO_2_ electrode sample, whereas the metallic Ga samples shown one intense peak at around 532.56 eV from Ga–O^52^ as indicated in Figure 8f. Here, based on O(1s) spectra, it is hard to identify LiGaO_2_ or Ga_2_O_3_ because of the similarity in their binding energy. The Li(1s) and Ga(3d) spectra can be used as solid evidence for the presence of LiGaO_2_ in the B-7 sample, which agrees to our previous observations from PXRD, Raman, and electrochemical analyses where the presence of LiGaO_2_ was confirmed too. The XPS survey for both LiGaO_2_ and metallic Ga included for comparison purpose as demonstrated in Figure 8g.

**Figure 9:**
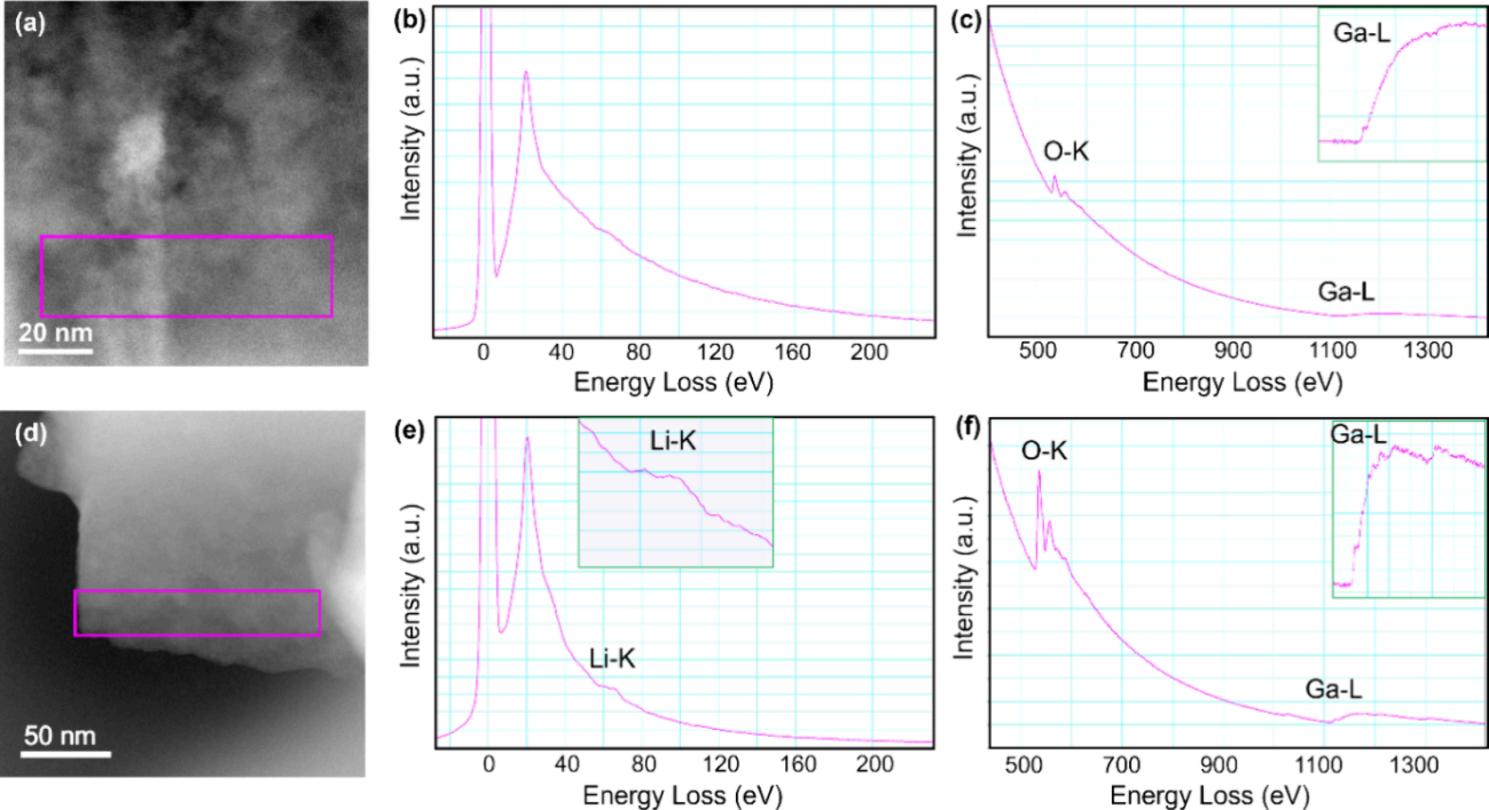
EELS spectra of the B-7 sample in different areas. (a) ADF-STEM image of the sample. (b) Low-loss EELS spectra, and (c) ionization edges corresponding to the O–K and Ga–L edges as indicated of selected area in panel a. The near-edge fine structure (ELNES) of Ga–L is enlarged (inset). (d) ADF-STEM image of the sample. (e) Low-loss spectra of the surface region, it shows the presence of Li. The ELNES of Li–K is enlarged (inset, f) corresponding O–K and Ga–L edges for the selected area as shown in panel d. The ELNES of the Ga–L peak is enlarged (inset).

Ga_2_O_3_ cannot be separated unambiguously from LiGaO_2_ through XPS, due to the coexistence of LiGaO_2_ and their similarities in Ga–O binding energies but could also not be separated from LiGaO_2_ at the length scale of SEM/EDX. To probe an even smaller length scale and decouple LiGaO_2_ form possible Ga_2_O_3_ signals, cryo-EELS measurements were conducted on the B-7 sample (charged to 4.5 V after the seventh cycle). A low-loss EELS spectrum from the bulk of this sample indicates the absence of Li in this area (Figure 9b). Figure 9c displays the O *K-*edge and Ga *L*-edge, enabling us to identify the composition of this region as Ga_2_O_3_,^53,54^ thus proving Ga_2_O_3_ formation from LiGaO_2_ decomposition above 3.5 V vs Li/Li^+^. Furthermore, in the surface area, as shown in Figure 9d–f, LiGaO_2_ was detected from its Li–K, O–K, and Ga–L signals. This proves the existence of Ga_2_O_3_ besides LiGaO_2_ on a length scale of a few hundred nm.

### Derived Reaction Mechanism for LiGaO_2_ Electrochemical Activity

**Figure 10:**
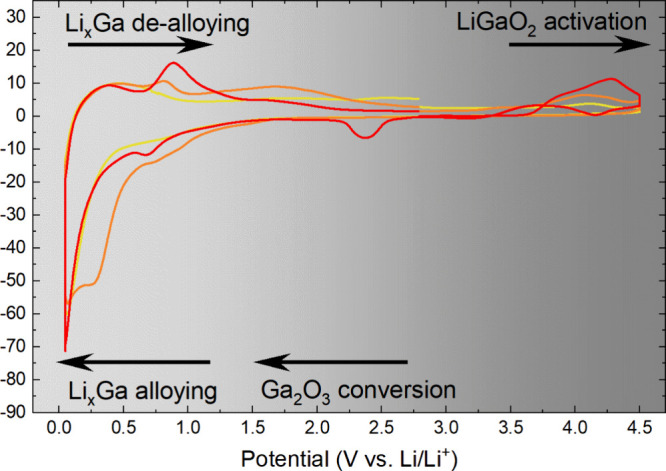
Overview of the identified reaction mechanism for LiGaO_2_ from 0.05 to 4.5 V vs Li/Li^+^.


Based on our observations from electrochemical analysis and *ex situ* Raman, XRPD, SEM/EDX, STEM/EELS, and synchrotron XPS analyses, we derive the following reaction mechanism for the electrochemical activity of LiGaO_2_ (Figure 10).
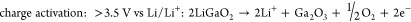
1

The first cycle starts from the OCV at 2.75 V vs Li/Li^+^. In the first charge, above 3.5 V versus Li/Li^+^, LiGaO_2_ is activated, meaning it starts to decompose. Li^+^ transfer from the positive LiGaO_2_ electrode to the negative Li-metal electrode is evident from the measured current. Here, Li^+^ is released into the electrolyte to be combined with one electron at the negative electrode, which goes through an external circuit from the positive to negative electrode. The remaining compound in the LiGaO_2_ electrode thus has to be oxidized to maintain the charge balance.

Ga as a group IIIA member can only have valence states as Ga^0^ or Ga^3+^. Formation of Ga^0^ at high potentials would contradict its calculated thermodynamic stability (Pourbaix diagram, Figure S4).^55−58^ Thus, Ga^3+^ is unlikely to change its valence state during the electrochemical oxidation reaction at high voltage, limiting the possible reaction product to Ga^3+^ containing oxide compounds. However, the formation of Li–Ga–O ternary compounds with a lower Li-stoichiometry than LiGaO_2_, such as LiGa_5_O_8_, does not agree with thermodynamic considerations of the Li–Ga–O stabilities at high voltage either. In fact, the thermodynamic calculations strongly suggest that Ga_2_O_3_ is formed.^55−58^ To form Ga_2_O_3_ from 2 LiGaO_2_, 1/2 O_2_ needs to be released to balance the reaction. This O_2_ evolution reaction (OER) is instantaneously coupled to the decomposition of LiGaO_2_ above 3.5 V vs Li/Li^+^ and will readily oxidize the other compounds in the electrode. The proposed OER during reaction (1) agrees with our observations in SEM/EDX, where large oxidations in the region of the binder matrix was observed in the charged sample after 7 cycles and where areas with high concentration of Ga were surrounded by an O-rich periphery, while Ga itself could not be separated from O even at the length scale of SEM/EDX. Finally, STEM/EELS revealed that Ga_2_O_3_ is present in the sample besides LiGaO_2_ at the nanometer length scale, thus unambiguously proving that LiGaO_2_ decomposes to Ga_2_O_3_ at potentials above 3.5 V vs Li/Li^+^, and Li being extracted electrochemically from the system.

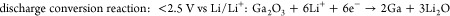
2
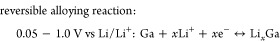
3

During discharge, measurable activity is detected below 2.5 V vs Li/Li^+^. The current increases steadily while going to lower potentials. Based on the previous observations for Ga_2_O_3_ formation in reaction 1, the reaction 2 during discharge is expected to arise from the Ga_2_O_3_ electrochemical conversion reaction, leading to metallic Ga and Li_2_O. The conversion reaction of Ga_2_O_3_ is well-known in the literature and has been demonstrated to lead to subsequent reversible alloying reactions that take place between 0.0 and 1.0 V vs Li/Li^+^.^36^ This agrees to the observed reversibility of the electrochemical reaction in our experiments below 1.0 V vs Li/Li^+^, for comparison see also Figure S5, Supporting Information.

The ideal electrochemical Li–Ga alloying reaction shows distinct electrochemical reactions at 0.14 V vs Li/Li^+^ during discharge and 0.78 and 0.86 V vs Li/Li^+^ during charge, Figure S6, Supporting Information. While striking differences can still be seen in terms of polarization and reversibility of the pure Li–Ga alloying reaction, the agreement with the alloying potential window of Li–Ga is obvious. Furthermore, our data agree very well with the Ga_2_O_3_ electrochemical activity,^36,37,47^ in terms of shapes and reversibility of the observed CV curves (Figure S5, Supporting Information). Furthermore, the reaction product in reaction 3 is metallic Ga, which has been clearly identified to be present in the charged sample after 7 cycles via XPS, in addition to Ga_2_O_3_ and LiGaO_2_.

The presence of LiGaO_2_ in all *ex situ* samples, as evidenced by XRPD, Raman spectroscopy, XPS, and EELS implies that, for the chosen experimental setup, LiGaO_2_ has not been fully activated during the recorded 7 cycles. This may be due to the rather large particle size of LiGaO_2_ that limits the electrochemical reaction to the surface of the LiGaO_2_ particles. Especially LiGaO_2_ itself does not provide sufficient electronic conductivity to free up electrons upon decomposition of bulk LiGaO_2_ in reaction 1. From the observed microstructural degradation through QPA based on the *ex situ* XRPD data, we can conclude that most of the activation of LiGaO_2_ takes place in the first charge. During the first discharge, Ga_2_O_3_ conversion is the major reaction, while in the subsequent charge and discharge cycles, the Li–Ga alloying and dealloying dominate.

The decomposition of LiGaO_2_ at electrochemical potentials >3.5 V vs Li/Li^+^ and the subsequent electrochemical reactions suggest that the presence of LiGaO_2_ at grain boundaries of Ga:LLZO electrolytes can subject full cell ASSLMB with Ga:LLZO electrolytes to short circuiting during cycling. In full cells, layered oxide positive electrodes (such as LiCoO_2_ or Li(Ni,Mn,Co)O_2_) are usually employed, which yield a cell potential of the ASSLMB at 4.2 V or higher.^1−3^ Cycling these cells will easily decompose LiGaO_2_ into Ga_2_O_3_. Since Ga_2_O_3_ has an electrochemical potential higher than metallic Li, Li^+^ will preferably react to Ga_2_O_3_ to form Ga and Li_2_O instead of reducing to Li metal at the negative electrode during the discharging process. Therefore, short circuiting of the ASSLMB will happen through the LiGaO_2_ phase even without physical contact to the metallic Li at the negative electrode. The experimental results thus highlight the importance of eliminating LiGaO_2_ formation at the grain boundaries of Ga:LLZO to realize its ASSLMB application.

## Conclusions

The study reports the electrochemical activity of LiGaO_2_, tested in a Li-ion type battery as positive electrode against Li metal as a negative electrode, with a Li^+^ conducting liquid electrolyte. We could show that LiGaO_2_ gets activated above 3.5 V vs Li/Li^+^, by decomposing to Ga_2_O_3_ under OER and the extraction of Li^+^ into the electrolyte, thus leading to the anodic reaction to Li metal at the negative electrode. The subsequent electrochemical reactions involve the reversible Li–Ga alloying and dealloying in the voltage range from 0.05 to 1 V vs Li/Li^+^ with significant capacities of up to 200 mAh g^–1^. The results have considerable implications for the use of full solid-state cells with Ga-doped garnet solid electrolytes, where LiGaO_2_ is known to coexist with the garnet main phase. Especially for the cycling conditions in full cells, usually above 3 V vs Li/Li^+^, LiGaO_2_ is expected to be activated and electrochemically reacted into Li–Ga alloy phases. Those reaction products will subject the solid electrolyte to cell failures through short circuits. The results highlight the importance of the knowledge-based design of solid electrolytes to realize high-performance solid-state batteries.

## Supporting Information Available

Additional experimental results and references: close-up view for XRD for all compounds; optical microscopy images for all compounds; table with reference structures in the quantitative phase analysis; SEM/EDX at cross section, Pourbaix diagram of Li–Ga–O; and comparison of LiGaO_2_ CV in this study with Ga_2_O_3_ CV from the literature, comparison of LiGaO_2_ CV in this study with Ga-metal CV as a control sample (PDF)
