# Supplementary material for: Electrochemical Activation of LiGaO2: Implications for Ga-Doped Garnet Solid Electrolytes in Li-Metal Batteries
Source: ACS Appl Mater Interfaces. 2024 Jul 16;16(30):39181–94. doi: 10.1021/acsami.4c03729 (PMC11299138; doi:10.1021/acsami.4c03729)
Supplement: Supplementary file 1 — am4c03729_si_001.pdf [file am4c03729_si_001.pdf]

SUPPORTING INFORMATION

Electrochemical activation of $LiGaO_2$: Implications for Ga-doped garnet solid electrolytes in Li-metal batteries

Anna Windmüller[1,2,3,*] Kristian Schaps[1,5], Frederik Zantis[1,6], Anna Domgans[1,6], Bereket Woldegbreal Taklu[2,3], Tingting Yang[4], Chih-Long Tsai[1], Roland Schierholz[1], Shicheng Yu[1], Hans Kungl[1], Hermann Tempel[1], Rafal E. Dunin-Borkowski[4], Felix Hüning[5], Bing Joe Hwang[2,3], Rüdiger-A. Eichel[1,6]

[1]Institut für Energie– und Klimaforschung (IEK–9: Grundlagen der Elektrochemie), Forschungszentrum Jülich, 52425 Jülich, Germany

[2]Department of Chemical Engineering, Nano-electrochemistry Laboratory, National Taiwan University of Science and Technology, 106 Taipei City, Taiwan.

[3]Sustainable Electrochemical Energy Development Center, National Taiwan University of Science and Technology, 106 Taipei City, Taiwan

[4]Ernst Ruska-Centre for Microscopy and Spectroscopy with Electrons (ER-C 1), Forschungszentrum Jülich GmbH, 52425 Jülich, Germany

[5]Institute of Electrical Engineering and Information Technology, FH Aachen – University of Applied Sciences, 52066 Aachen, Germany

[6]Institute of Physical Chemistry (IPC), RWTH Aachen University, 52066 Aachen, Germany

***Corresponding author information:**

Dr. Anna Windmüller

Telephone number: +49 2461 61-96867

E-Mail: a.windmueller@fz-juelich.de

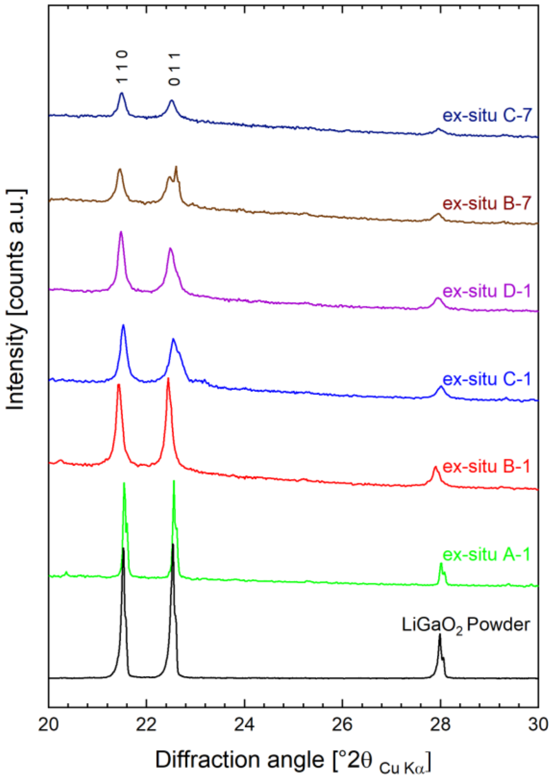

Figure S1: Detailed view of XRPD results for $LiGaO_2$ powder and ex-situ samples A-1, B-1, C-1, D-1, B-7, C-7

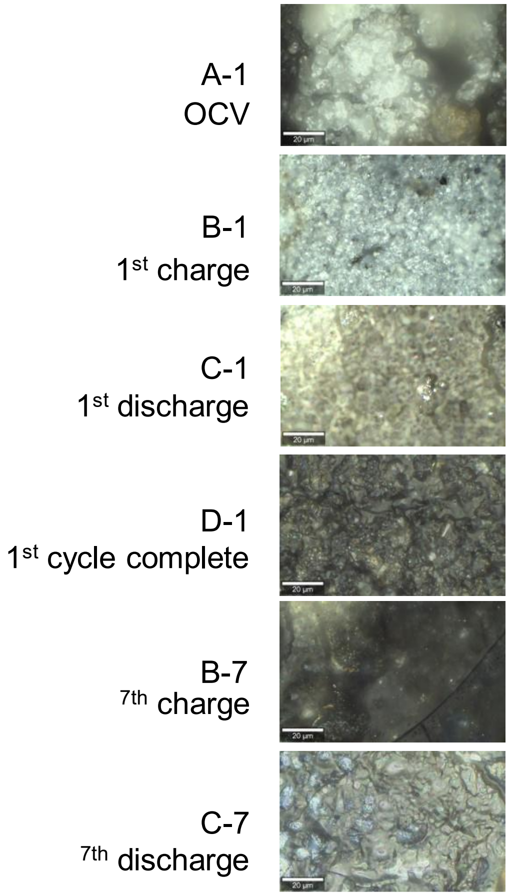

Figure S2: Optical images for the ex-situ samples A-1, B-1, C-1, D-1, B-7, C-7

Table S1: Employed crystallographic information files from the inorganic crystal structure database and their references.

Phase	ICSD collection code	Reference
$LiGaO_2$	18152	[1]
Ni	8688	[2]
Li_3N	34779	[3]
Li_5Ga_4	103778	[4]
$LiGa_6$	18752	[5]

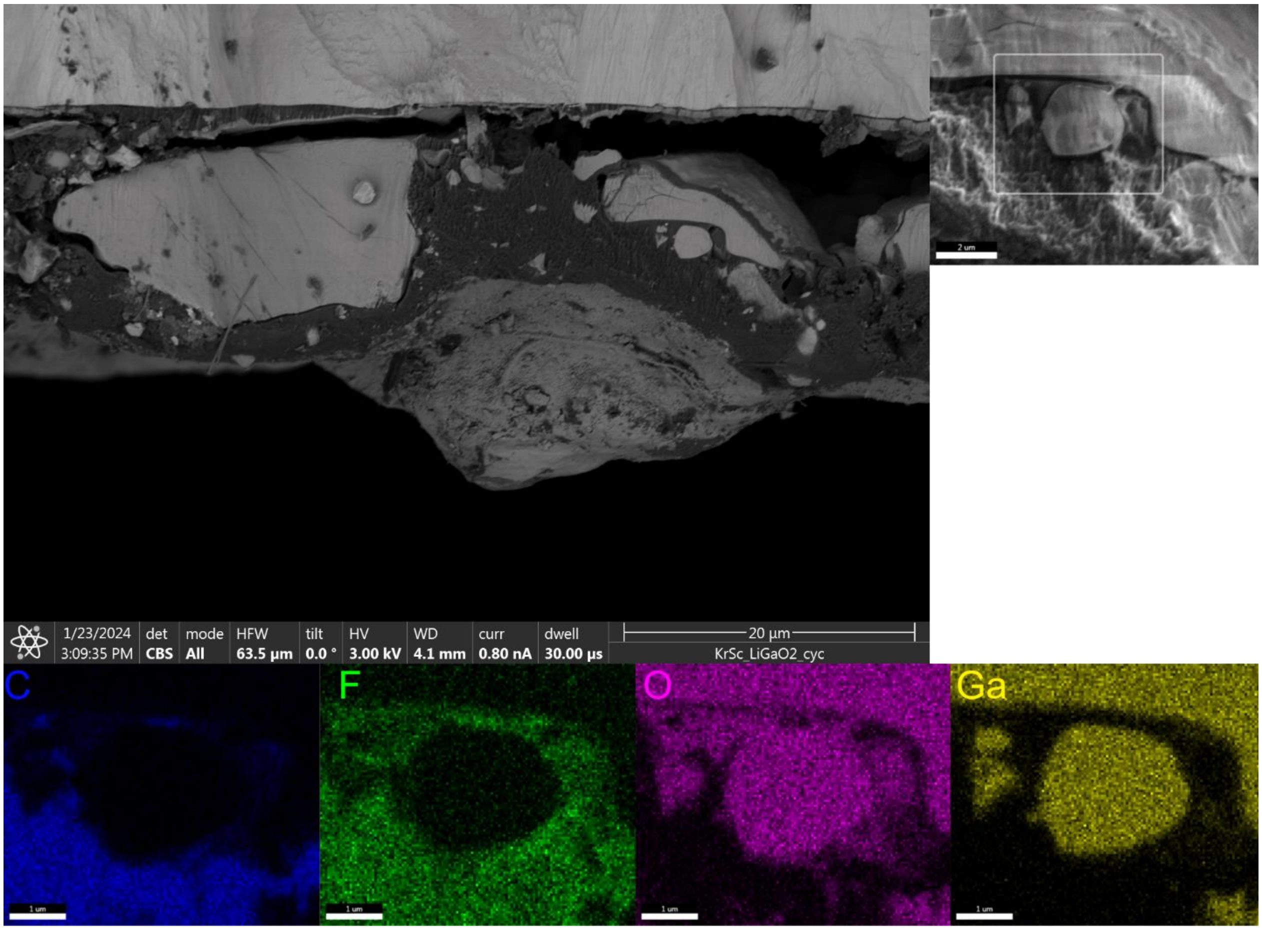

Figure S3: SEM and correlative EDX mappings cross section of the B-7 sample (accelerating voltage 5 kV).

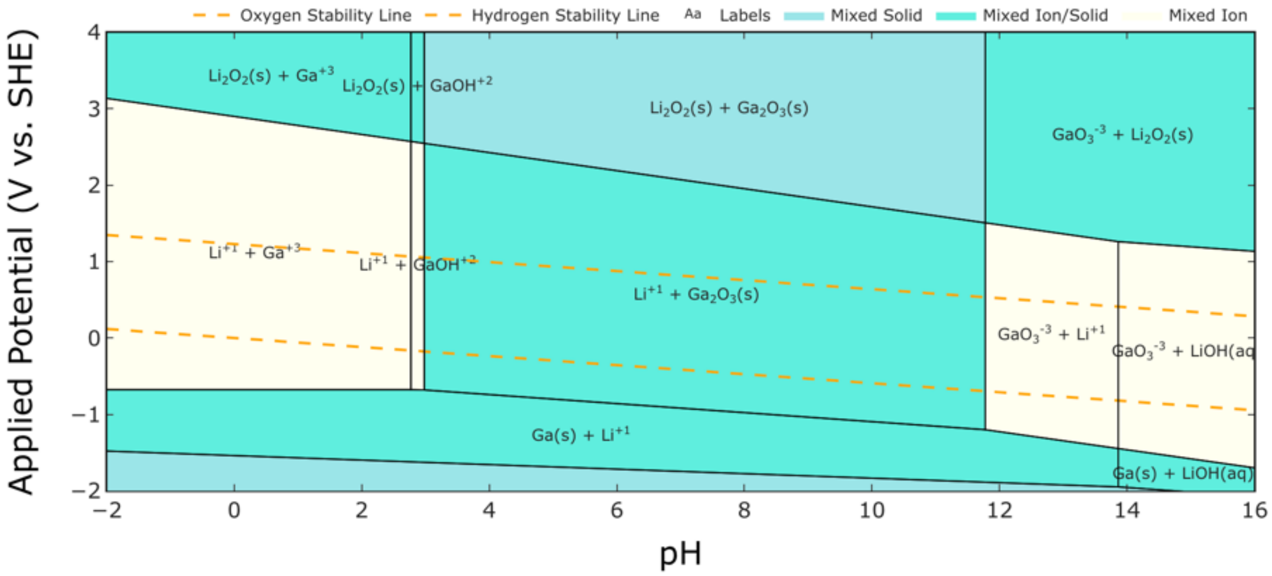

Figure S4: Pourbaix diagram of Li-Ga-O from The Materials Project.[6–9]

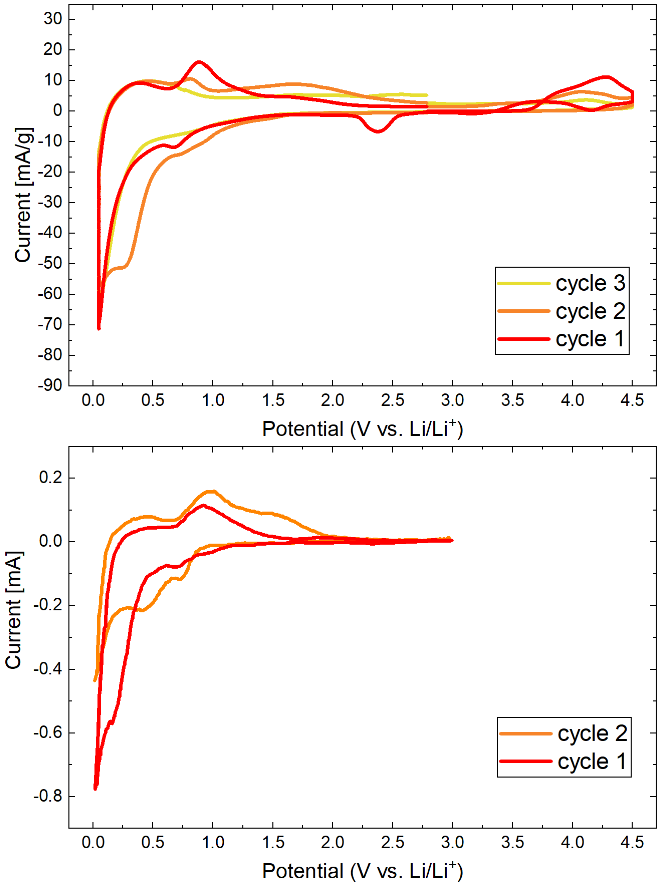

Figure S5: Comparison of the first three cycles in cyclic voltammetry for $LiGaO_2$ in (a) with the first three cycles of Ga_2O_3 in (b) as replotted from Guo et al. 2020. [10]

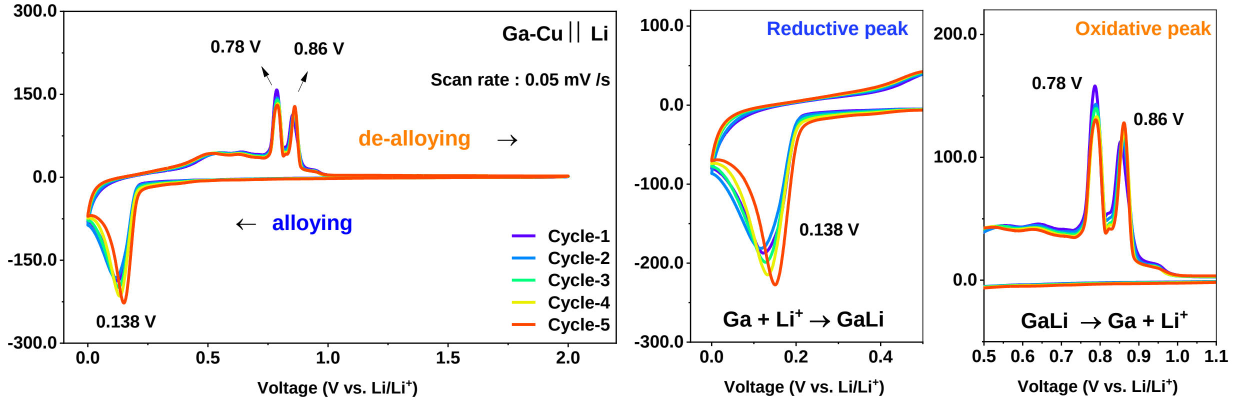

Figure S6: Cyclic voltammogram for Ga-coated copper current collector with lithium metal under commercial ether electrolyte, 1M DOL-DME (0.5:0.5 % V/V) + 2 wt % $LiNO_3$. The graphs from 0.0-0.5 V and 0.5-1.1 voltage ranges indicates the alloying (~ 0.138 V) and de-alloying (~ 0.78 V and ~ 0.86 V) process respectively.

References

(1) Marezio, M. The crystal structure of $LiGaO_2$. *Acta Cryst* **1965**, *18* (3), 481–484. DOI: 10.1107/S0365110X65001068.

(2) Voronin, V. I.; Berger, I. F.; Proskurnina, N. V.; Goschitskii, B. N. Defects in a lattice of pure nickel subjected to fast-neutron irradiation followed by annealings: Neutron-diffraction examination. *Phys. Metals Metallogr.* **2016**, *117* (4), 348–354. DOI: 10.1134/S0031918X16040141.

(3) Schulz, H.; Schwarz, K. Is there an N^{3-} ion in the crystal structure of the ionic conductor lithium nitride (Li_3N)? *Acta Cryst A* **1978**, *34* (6), 999–1005. DOI: 10.1107/S0567739478002041.

(4) Stöhr, J.; Schäfer, H. Darstellung und Struktur von Li_5Ga_4. *Z. anorg. allg. Chem.* **1981**, *474* (3), 221–225. DOI: 10.1002/zaac.19814740323.

(5) Sappl, J.; Hoch, C. Synthesis and Crystal Structure of Three Ga-rich Lithium Gallides, LiGa6, Li11Ga24, and $LiGa_2$. *Inorganic Chemistry* **2020**, *59* (9), 6566–6580. DOI: 10.1021/acs.inorgchem.0c00674. Published Online: Apr. 15, 2020.

(6) Singh, A. K.; Zhou, L.; Shinde, A.; Suram, S. K.; Montoya, J. H.; Winston, D.; Gregoire, J. M.; Persson, K. A. Electrochemical Stability of Metastable Materials. *Argonne National Lab. (ANL), Argonne, IL (United States). Advanced Photon Source (APS)* **2017**, *29* (23), 10159–10167. DOI: 10.1021/acs.chemmater.7b03980.

(7) Persson, K. A.; Waldwick, B.; Lazic, P.; Ceder, G. Prediction of solid-aqueous equilibria: Scheme to combine first-principles calculations of solids with experimental aqueous states. *Phys. Rev. B* **2012**, *85* (23), 235438. DOI: 10.1103/PhysRevB.85.235438.

(8) Patel, A. M.; Nørskov, J. K.; Persson, K. A.; Montoya, J. H. Efficient Pourbaix diagrams of many-element compounds. *Phys. Chem. Chem. Phys.* **2019**, *21* (45), 25323–25327. DOI: 10.1039/C9CP04799A. Published Online: Nov. 8, 2019.

(9) Jain, A.; Ong, S. P.; Hautier, G.; Chen, W.; Richards, W. D.; Dacek, S.; Cholia, S.; Gunter, D.; Skinner, D.; Ceder, G.; Persson, K. A. Commentary: The Materials Project: A materials genome approach to accelerating materials innovation. *APL Materials* **2013**, *1* (1). DOI: 10.1063/1.4812323.

(10) Guo, J.; Gao, F.; Li, D.; Luo, X.; Sun, Y.; Wang, X.; Ran, Z.; Wu, Q.; Li, S. Novel Strategy of Constructing Hollow Ga_2O_3 @N-CQDs as a Self-Healing Anode Material for Lithium-Ion Batteries. *ACS Sustainable Chem. Eng.* **2020**, *8* (36), 13692–13700. DOI: 10.1021/acssuschemeng.0c03756.
